# EQRbot: A chatbot delivering EQR argument-based explanations

**DOI:** 10.3389/frai.2023.1045614

**Published:** 2023-03-23

**Authors:** Federico Castagna, Alexandra Garton, Peter McBurney, Simon Parsons, Isabel Sassoon, Elizabeth I. Sklar

**Affiliations:** ^1^School of Computer Science, University of Lincoln, Lincoln, United Kingdom; ^2^Department of Informatics, King's College London, London, United Kingdom; ^3^Department of Computer Science, Brunel University London, London, United Kingdom; ^4^Lincoln Institute for Agri-Food Technology, University of Lincoln, Lincoln, United Kingdom

**Keywords:** argument schemes, computational argumentation, chatbot, explainability, decision-support systems, healthcare, XAI

## Abstract

Recent years have witnessed the rise of several new argumentation-based support systems, especially in the healthcare industry. In the medical sector, it is imperative that the exchange of information occurs in a clear and accurate way, and this has to be reflected in any employed virtual systems. Argument Schemes and their critical questions represent well-suited formal tools for modeling such information and exchanges since they provide detailed templates for explanations to be delivered. This paper details the EQR argument scheme and deploys it to generate explanations for patients' treatment advice using a chatbot (EQRbot). The EQR scheme (devised as a pattern of Explanation-Question-Response interactions between agents) comprises multiple premises that can be interrogated to disclose additional data. The resulting explanations, obtained as instances of the employed argumentation reasoning engine and the EQR template, will then feed the conversational agent that will exhaustively convey the requested information and answers to follow-on users' queries as personalized Telegram messages. Comparisons with a previous baseline and existing argumentation-based chatbots illustrate the improvements yielded by EQRbot against similar conversational agents.

## 1. Introduction

Artificial Intelligence constitutes a powerful means when deployed for assisting people in making well-informed decisions. Such assistance is delivered as a set of recommendations on which a human, who is interacting with the AI-based system, has the final word. In the healthcare sector, decision support systems (DSS) prove to be especially useful since they mostly present: time-saving virtual assistance for practitioners; help for patients in self-managing their health conditions; better documentation, retrieval and presentation of data (which, as stated in Fairweather et al. (2020), is still required to be reliable by showing that its provenance is non-repudiable); and, finally, a substantial cost saving due to the partial automation and optimization (while preferring cheaper, but still effective, treatment options) of the workflow (Sutton et al., 2020). Several DSS employ advanced machine learning algorithms as their main AI reasoning mechanism, although they do not seem to provide robust evidence of improved diagnostic performance in clinical environments (Vasey et al., 2021). Other DSS employ computational argumentation instead as their AI reasoning mechanism. Indeed, as highlighted by Lindgren et al. (2020), the handling of inconsistent and conflicting knowledge is a common feature in medical decision-making processes when the opinions of several medical experts are solicited with regard to specific cases. Arguments can reflect the opinion of a single practitioner, of a general/local medical guideline or even represent the viewpoint of a patient concerning a particular treatment. As an example of argumentation-driven clinical DSS (henceforth cDSS), the authors of Kökciyan et al. (2021) model medical recommendations *via* meta-level arguments that makes it possible to determine the ground on which the object-level arguments are justified or preferred. The work of Cyras et al. (2018) moves, instead, toward the creation of a cDSS that employs the structured argumentation formalism of ABA^+^ (stemming from the Assumption-Based Argumentation framework originally described in Bondarenko et al., 1997) for automated reasoning with conflicting clinical guidelines, patients' information and preferences. Multiple studies have also been conducted in the field of cDSS considering patients suffering from multimorbidities (as in Oliveira et al., 2018 and Chapman et al., 2019). Although the results thus far achieved have mostly been positive, in Bilici et al. (2018) the authors emphasize the need for further investigations regarding considerations of shared decisions, patients' preferences and social contexts, and a broader range of drug interactions (including food-drug interactions). Argumentation-based cDSS have been devised also in this specific research area: the CONSULT project (outlined in papers such as Essers et al., 2018; Balatsoukas et al., 2019; Kökciyan et al., 2019) introduces a data-driven decision support tool to help patients with chronic conditions manage their multimorbidities in collaboration with their carers and the health care professionals who are looking after them.

The drive to overcome ethical issues involving AI-based systems, along with distrust from their users, constitutes the reason for the recent interest in the field of Explainable AI (XAI). The idea is that the trustworthiness of AIs can be improved by building more transparent and interpretable tools capable of: explaining what the system has done, what it is doing now and what it is going to do next while disclosing salient information during these processes (Bellotti and Edwards, 2001). Nevertheless, Vilone and Longo (2021) point out that there is no general consensus upon an unambiguous definition of explanations and their essential properties. Drawing from social sciences studies, Miller (2019) identifies specific features that could help characterize explanations, all of which converge around a single conclusion: explanations are *contextual*. Similarly, Bex and Walton (2016) consider explanations as speech acts, differentiated by context from other locutions, used to help *understand* something. More precisely, explanations are a transfer of understanding from one party to another, where understanding is intended as common knowledge” shared between those parties. That said, there still remain many active issues concerning XAI. In Gunning et al. (2019), the authors present a (non-exhaustive) list of these challenges, that includes topics such as: accuracy vs. interpretability, the use of abstractions to simplify explanations or prioritizing competencies over decisions. Another problem is related to the end-user who is meant to receive the explanation. Indeed, the explainee might be an individual with a specific background. Taking into account the different knowledge and clarification needs of each target user group will ensure the generation of more compelling explanations. From this perspective, it is interesting to notice that the research presented in Antaki and Leudar (1992), and more recently in Cyras et al. (2021), propose an account of explanations that is primarily argumentative. Similarly, the survey of Vassiliades et al. (2021) concludes that using argumentation to justify why an event started, or what led to a decision, can enhance explainability. These intuitions are also backed by McBurney and Parsons (2021), where it is suggested that AI systems should adopt an argumentation-based approach to explanations. The advocated approach points toward Douglas Walton's Argument schemes (AS), thoroughly discussed in Walton et al. (2008).

The paper is structured as follows. Starting from a brief introduction of the required background notions in Section 2, we will propose a new dialectical tool for delivering cDSS recommendations: the EQR scheme, its corresponding critical questions, and the role that such a model plays in providing explanation within the clinical setting (Section 3). Section 4 articulates its implementation in the context of the CONSULT system, whereas Section 5 describes the chatbot (EQRbot) and its internal architecture. The bot conveys information starting from an instantiated EQR scheme around which pivots any additional answer to follow-on users' questions. Finally, Sections 6 and 7 provide a discussion and conclusion, respectively.

### 1.1. Contributions

The research outlined in this paper presents several original contributions. Expanding on the previous work of Castagna et al. (2022) that sketched the novel EQR scheme, we are going to (1) provide a more detailed description of the EQR scheme. Such a formal structure emerges as an effective model for conveying practical and theoretical information yielded as consequences of a presumptive reasoning formalization involving acting upon an expert opinion. The EQR scheme herein proposed proves to be particularly suited in concentrating relevant knowledge within a single explanation. For this reason, we devise (2) an implementation in the form of a chatbot (EQRbot) integrated into the CONSULT system. This bot delivers tailored EQR-based recommendations to patients, helping them self-managing their conditions. These recommendations also embed an additional layer of information: the rationale behind the instantiated scheme acceptability (i.e., its evaluation according to the considered argumentation framework). Finally, the EQRbot main procedure draws from our third contribution: (3) an algorithm for computing and delivering explanations, of which we provide (4) a formal analysis of the performance.

## 2. Background

The following background covers a concise summary of computational argumentation, along with a short overview of how argument schemes (and their clinically specialized version) have been employed in the literature to deliver medical explanations. The introduced formal definitions and models will prove useful in the next sections.

### 2.1. Computational argumentation

Informal studies on argumentation are underpinned by a rich literary heritage, but it is only in the past decades that logic-based models of argumentation have been intensively investigated as core components of AI-driven and Multi-Agent Systems (Chesnevar et al., 2000; Bench-Capon and Dunne, 2007). The seminal work conducted in Dung (1995) has been the starting point for most of the recent interest and research in the field of abstract argumentation and its argumentative characterizations of non-monotonic inferences. Indeed, the main strength of his approach is the simple and intuitive use of arguments as a means to formalize non-monotonic reasoning while also showing how humans handle conflicting information in a dialectical way. In a nutshell, the idea is that correct reasoning is related to the admissibility of a statement: the argument is acceptable (i.e., justified) only if it is defended against any counter-arguments. The core notion of Dung's abstract approach revolves around the definition of an argumentation framework, that is a pair AF = 〈AR, *attacks*〉, where AR is a set of arguments, and ‘*attacks'* is a binary relation on AR, i.e., *attacks* ⊆ AR × AR, such that *attacks*(*X, Y*) denotes the conflict existing between an argument *X* and its target *Y*. In the same paper, the author proposes also different semantics to capture alternative (skeptical or credulous) types of reasoning:

Definition 1 (*Argumentation semantics*). *Let* AF = 〈AR, *attacks*〉*, and* S ⊆ *AR be a set of arguments:*

- S is *conflict free* iff ∀X,Y∈S: ¬*attacks*(*X, Y*);
- *X* ∈ AR is acceptable w.r.t. S iff ∀ *Y* ∈ AR such that *attacks*(*Y, X*): ∃Z∈S such that *attacks*(*Z, Y*);
- S is an *admissible* extension iff X∈S implies *X* is acceptable w.r.t. S;
- An admissible extension S is a *complete* extension iff ∀*X* ∈ AR: *X* is acceptable w.r.t. S implies X∈S;
- The least complete extension (with respect to set inclusion) is called the *grounded extension*;
- A maximal complete extension (with respect to set inclusion) is called a *preferred extension*.

As anticipated, AFs represent general frameworks capable of providing argumentative characterizations of non-monotonic logics.^1^ That is to say, given a set of formulae Δ of some logical language *L*, AFs can be instantiated by such formulae. The conclusions of justified arguments defined by the instantiating Δ are equivalent to those obtained from Δ by the inference relation of the logic *L*. These instantiations paved the way for a plethora of different studies concerning the so-called “structured” argumentation (as opposed to the abstract approach). Among these, Besnard and Hunter (2008), Modgil and Prakken (2013), and Toni (2014) describe a formalization of arguments that follows the same model of the Argument Schemes introduced in Walton et al. (2008). That is to say, arguments are typically used to advocate a claim based on the premises put forward as evidence to support such a claim.

### 2.2. Argument schemes and explanations in clinical settings

Argument schemes have been extensively investigated and employed in the AI literature as a way to directly convey presumptive reasoning in multi-agent interactions (for example, Atkinson et al., 2006; Tolchinsky et al., 2012; Grando et al., 2013). Each AS is characterized by a unique set of critical questions (**CQ**s), rendered as attacking arguments, whose purpose is to establish the validity of the scheme instantiations. This generates an argumentation framework that can then be evaluated according to one of the semantics described in Dung (1995). Such evaluation embeds the rationale for choosing an argument over another, meaning that justified instantiations of schemes can be employed for conveying explanations. The use of argument schemes for providing explanations is, indeed, not unusual, especially in the clinical setting. In Shaheen et al. (2021), the authors introduce the *Explain Argument Scheme*, which models explanations based on the reasons, types (of reasons) and levels (of abstraction) and shows a (pro or con) rationale for giving a particular drug to a patient. The work presented in Sassoon et al. (2019), Kökciyan et al. (2020), and Sassoon et al. (2021) harnesses *Explanation Templates* that differ according to the reasoning and argument scheme represented and include placeholders for the actual instantiated variables specific to a given application of the scheme. Formally:

Definition 2 (*Argument Scheme*). AS = 〈Prem, Con, Var〉 *denotes an argument scheme, where* Prem *is a set of premises*, Con *is the conclusion, and* Var *is the set of variables used in the argument scheme*.

Definition 3 (*Explanation Template*). *Let* AS *be an argument scheme* (*as per Definition* *2*)*, and* txt *be a natural language text that includes elements from* Var*. Then, an Explanation Template for* AS *can be rendered as the tuple* Expl_AS_ = 〈AS,txt〉.

Definition 4 (*Explanation*). *An explanation is a tuple* 〈Expl_AS_, AS_*i*_〉 *such that* Expl_AS_ *is the explanation template introduced in Definition* *3*, AS_*i*_ *is an acceptable* (*as per Definition* *1*) *instantiation of* AS *with respect to some AF, and every variable in* txt *of* Expl_AS_ *is instantiated by the corresponding element in* AS_*i*_.

Intuitively, Explanation Templates are engineered to be adaptive toward the circumstance of their employment and thus generate tailored explanations. That is to say, argument schemes model stereotypical patterns of reasoning in different generic situations, increasing their versatility of usage thanks to a number of integrated variables. Leveraging those variables, Definition 3 depicts formal structures that further enhance their flexibility by considering specific natural language snippets concerning the current context. These structures account then for explanations that enjoy the *contextuality* property (one of the most relevant features of explanations according to Miller, 2019), while they also acknowledge the end-users' different knowledge, understanding capability, and clarification needs.

### 2.3. Clinically specialized argument schemes

In order for a cDSS to provide the appropriate medical suggestions, explanation templates have previously been mapped to the *Argument Scheme for Proposed Treatment* (ASPT) (Sassoon et al., 2019, 2021; Kökciyan et al., 2020). Introduced in Kokciyan et al. (2018), ASPT derives from the *Argument Scheme for Practical Reasoning* as presented in Atkinson and Bench-Capon (2007). It instantiates an argument in support of a possible treatment, given the facts *Ft* about the patients and the goal *G* to be achieved.

**ASPT**

*Premise* : Given the patient's fact Ft

*Premise* : In order to realize goal G

*Premise* : Treatment T promotes goal G

*Conclusion* : Treatment T should be considered

As with each argument scheme, ASPT is accompanied by a series of critical questions that serve to assess the efficacy of the proposed treatment. In Sassoon et al. (2021), some of these questions are modeled as clinical specializations of existing argument schemes (listed in Walton et al., 2008) and cover particular aspects of the suggested treatment, such as *AS from Patient Medical History, AS from Negative Side Effect* and *AS for Contraindications*.

## 3. Methods: Providing explanations *via* the EQR argument scheme

### 3.1. EQR argument scheme

Devised as a model of **E**xplanation-**Q**uestion-**R**esponse agents interactions sketched in McBurney and Parsons (2021), the EQR argument scheme draws from the *AS for Practical Reasoning* (the variation of the AS presented in Walton (1996) as characterized in Atkinson and Bench-Capon, 2007) and the *AS from Expert Opinion* (Walton, 1997). The underlying idea is to merge the knowledge elicited by those two formal patterns in a single scheme that would then yield the advantage of concentrating and synthesizing the same amount of information in a unique data structure that may be queried more conveniently. That is to say, the purpose of the EQR scheme is to formalize the consequences arising (and the presumptive reasoning leading to them) by acting upon a specific expert opinion. A reference to such authority provides the rationale that justifies the conclusion of the argument, also leaving chances of inquiry for more detailed explanations.

The proposed scheme assumes the existence of:

- A finite set of knowledgeable experts, called *Experts*, denoted with elements E, E', etc. Experts are deemed knowledgeable if they can somehow prove their competencies (e.g., years of experience, professional achievements, research publications).
- A finite set of disciplinary fields of expertise, called *Fields*, denoted with elements F, F', etc.
- A finite set of propositions, called *Opinions*, denoted with elements *α*, *β*, etc. Each member represents the viewpoint of an expert with regard to a specific topic.
- A finite set of propositions, called *Prop*, denoted with elements A, B, etc.
- A finite set of states, called *States*, denoted with elements R, S, etc. Every member describes a specific state of the world and corresponds to an assignment of truth values {Truth,False} to every element of *Prop*.
- A finite set of *Values* denoted with elements v, w, etc. This category includes both positive (i.e., constructive, such as wellbeing, altruism, integrity, etc.) and negative (i.e., non-constructive, such as dishonesty, manipulation, greed, etc.) values.
- A function acting_upon that maps each element of *Opinions* to a member of *States*.

**EQR**

*Premise* : In the current state R

*Premise* : acting upon *α* (from an expert E in a field F)

*Premise* : will result in a new state S

*Premise* : which will make proposition A true (alternatively, false)

*Premise* : which will promote some value v

*Conclusion* : Acting upon the opinion *α* should make proposition

A true (false) and entail value v

Intuitively, starting from the current circumstance R and acting upon the opinion asserted by a competent expert in the relevant field, the agent instantiating the scheme wishes to attain A (or not A) and the actual reason for it (value v), along with the entailed consequences, whether they are desired or not (new state S). As an example of expert opinion, consider an architect asserting that, according to her recent evaluation, the nearby bridge requires immediate maintenance to prevent its collapse. In this case, by acting upon such an opinion, the practical intervention of specialized workers will change the state of the world into a new state where the bridge is no longer precarious (promoting the safety value).

The EQR scheme is accompanied by specifically designed critical questions:

(**EQR.CQ1**) Is E the most knowledgeable expert source?
(**EQR.CQ2**) Is E trustworthy?
(**EQR.CQ3**) Is E an expert in the field F that *α* is in?
(**EQR.CQ4**) Would acting upon *α* imply A (or not A)?
(**EQR.CQ5**) Are there alternative experts' opinions that can be acted upon to imply A (or not A)?
(**EQR.CQ6**) Would acting upon *α* entail contradictory propositions?
(**EQR.CQ7**) Is A consistent with what other experts assert?
(**EQR.CQ8**) Is *α* based on the (facts expressed by) state R?
(**EQR.CQ9**) Is F the most relevant disciplinary field to A given the (facts expressed by) state R?
(**EQR.CQ10**) Would acting upon *α* promote a negative value?

Following an approach akin to Sassoon et al. (2021), we can model each of the above critical questions into corresponding argument schemes. Each of these additional argument schemes may have its respective critical questions. However, we are omitting them since a full list of CQs for every possible argument scheme elicited by the critical questions of EQR is out of the scope of the current paper. For simplicity, we are going to outline only three of such templates.

#### 3.1.1. AS for expert reliability (ASEXP)

**ASEXP**

*Premise* : Given a set of knowledgeable experts

*Premise* : E is more trustworthy and knowledgeable than any other experts

*Conclusion* : E should be considered the most reliable expert

The *AS for Expert Reliability* fleshes out why a proficient source should be regarded as the most reliable (i.e., the most knowledgeable and trustworthy) in a group of several experts (if any). This is connected with and models EQR.CQ1-CQ2. Notice that here we are assuming a hierarchy of experts based on their reliability achieved by a preliminary probing of the ASEXP scheme instantiation (through its respective CQs) and the available professionals in the set of *Experts* that informs the EQR scheme instantiation. As an example, we could envisage a team of archaeologists at different stages of their careers. Everyone is considered an expert with several years of experience in their competence area. However, among them, there is a person (E) who has published more research articles and has participated in more archaeological excavations than any other member of the examined group of professionals (most knowledgeable). In addition, E has also diligently conducted the role of treasurer in each past expedition he took part in (trustworthy). Therefore, E can be deemed as the most reliable expert within those present. Observe that the same result will also occur if E is the only element of the considered set. Anticipating our implementation of the scheme within the CONSULT cDSS, let us also present another example that considers, like the aforementioned system, only clinical guidelines as Experts. This may yield an ASEXP instantiation where the World Health Organization (WHO) and other local practices are compared. WHO guidelines^2^ (E), informed by several global professionals in a multitude of medical areas, result in the most knowledgeable source of expertise if measured against any other guidances based upon the proficiency of smaller (often not international) local practitioners teams, as occurs for hospital guidelines. The formers also emerge as the most trustworthy guidances since they are regularly inspected by a specific review committee composed of appropriately trained staff members. As such, E can be regarded as the most reliable expert among those present.

#### 3.1.2. AS for relevant field of expertise (ASF)

**ASF**

*Premise* : Given a set of disciplinary fields of expertise

*Premise* : Given the current state R

*Premise* : Given a goal to achieve G

*Premise* : F yields more connections, with respect to R and G,

than any other fields

*Conclusion* : F should be considered the most relevant disciplinary field

The *AS for Relevant Field* provides the rationale for identifying the most relevant field, with respect to the current state of affairs R and a goal to achieve G, among a set of different disciplinary fields of expertise. This AS is correlated with and models EQR.CQ9. Once again, we are assuming a hierarchy of fields of expertise, based on their relevance over R and G, achieved by a preliminary probing of the ASF scheme instantiation (through its respective CQs) and the available elements in the set of *Fields* that informs the EQR scheme instantiation. As an example, consider R to be a state where a pandemic has spread to a whole country. To deal with such an emergency and promote people's health (G), we should probably resort to epidemiology as a more relevant field of expertise rather than, say, oncology or neurology. That is because the former can be deemed as having more connections with R and G, hence proving to be more relevant than the latter.

#### 3.1.3. AS for alternatives options (ASO)

**ASO**

*Premise* : Given a set of alternative options

*Premise* : Given circumstance C

*Premise* : Option O does not cause complications in circumstance C

*Conclusion* : O should be selected

The *AS for alternative Options* examines the reasons why a specific option, given a particular circumstance C, should be selected among a set of alternative options. This AS is correlated with and models EQR.CQ5. As an example, we can picture a man that needs to testify in court for a robbery he witnessed. Unfortunately, he also knows the thief. The man is now required to choose between producing a deposition that will incriminate his acquaintance or lying about having witnessed the crime at all. However, since perjury is a prosecutable criminal offense, telling the truth proves to be the only option that does not cause legal complications. As such, the witness will select the former alternative.

### 3.2. EQR and explanations in medical setting

Intuitively, the EQR scheme can display a large number of information bits to an *explainee* when looking for clarifications about a proposed treatment. Notice indeed that the EQR scheme can encompass ASPT such that it renders: (i) the treatment *T* as the expert's opinion *α* (from an expert E in a field F); (ii) the patient fact *Ft* as part of the current state *R* and (iii) the goal to be realized *G* as proposition *A*. That is to say, by embedding ASPT into the EQR scheme, it will be possible to give more opportunities for inquiry to an agent seeking clinical recommendations. Certainly, in this way, further aspects can be interrogated and this can lead to more satisfactory (and complete) explanations. For example, the additional data comprised in the current state R, the connected field of expertise F, the immediate consequence S entailed by the proposed treatment, or the value v conveyed by the truth-value of A, all of these are elements that can be interrogated by the patients. In particular, knowing the source of the recommendation E (in the remainder of the paper, this will correspond to the chosen clinical guideline) may boost the patient's trust in the explainer and the advised medical care plan. Moreover, the rationale behind the provided explanations can be further investigated (resulting in additional, more detailed, explanations) thanks to the extra information supplied by the answers to each critical question and corresponding argument that informs valid instantiations of the EQR scheme (and the incorporated ASPT). This entails that the same CQs that challenges ASPT will also question instantiations of the EQR scheme when deployed for medical recommendations. For example, the CQs concerning the presence of contraindications and negative side effects within the proposed treatment (that structure *AS for Contraindications* and *AS from Negative Side Effect* and in the work of Sassoon et al., 2021) will revise the previously introduced *AS for alternative Options* in a clinically specialized form. The resulting *AS for alternative Clinical Options* (ASCO) describes the reasoning pattern that elicits the choice of a specific harmless treatment for a patient, considering her health conditions. Indeed, the selection of the recommended remedy is informed by the subject's health record: it thus strictly avoids any potentially dangerous medication. As an example, depict R as the state that includes a patient suffering from a bacterial chest infection. There are three available antibiotics that can treat such a disease in the current state R: amoxicillin^3^, cefalexin^4^, and azithromycin.^5^ According to the information documented by the subject's medical facts (Ft) embedded in R, the patient is particularly sensitive to joint and muscle pain, which is listed among the amoxicillin side effects. Furthermore, azithromycin should be avoided due to its contraindications for people affected by heart problems, as, suppose, is our virtual subject. On the other hand, cefalexin (T) has already been administered to the patient in the past without resulting in any dangers or complications. As such, the latter is the treatment that should be recommended to cure the infection.

An EQR Explanation Template is then determined as in Definition 3, although it employs the EQR scheme rather than a generic AS. Similarly, we can formalize an instance of such a template as:

Definition 5 (*EQR Explanation*). *An* EQR *explanation is a tuple* 〈Expl_EQR_, EQR_*i*_〉 *such that* Expl_EQR_ *is the explanation template for the* EQR *scheme*, EQR_*i*_ *is an acceptable* (*as per Definition* *1*) *instantiation of the* EQR *scheme with respect to some AF, and every variable in* txt *of* Expl_EQR_ *is instantiated by the corresponding element in* EQR_*i*_.

**ASCO**

*Premise* : Given a set of alternative treatments

*Premise* : Given the current state R

*Premise* : Considering the patient's fact Ft (subsumed in R),

treatment T does not cause contraindication nor side effects

*Conclusion* : T should be recommended

Example 1. Suppose that we have an acceptable (as per Definition 1) clinical instantiation of the *EQR* scheme, informed by its critical questions and a specific knowledge base. Assume also that the scheme variables Var = {*R, E, F*, *α*, *S, A, v*} are equivalent to the following:

[*R*]: *the patient's previous health record and the current fever and headache (due to COVID-19)*
[*E*]: *the NICE guidelines*^6^
[*F*]: *medical management of COVID-19*
[*α*]: *the administering of paracetamol*
[*S*]: *the reduction of fever and headache*
[*A*]: *controlling the negative effect of the COVID-19 virus*
[*v*]: *the patient's wellbeing*

Finally, let txt be the natural language text: *Given* [*R*]*, the expertise of* [*E*] *in the field of* [*F*] *indicates* [*α*] *as an effective treatment. This should lead to* [*S*] *which will bolster the goal of* [*A*] *and promote* [*v*]”. Then, the actual *EQR* Explanation would be:

“*Given* ***the patient's previous health*** ***record and the current fever and headache (due to COVID-19)****, the expertise of* ***the NICE guidelines*** *in the field of* ***medical management of COVID-19*** *indicates* ***the administering of paracetamol*** *as an effective treatment. This should lead to* ***the reduction of fever and headache*** *which will bolster the goal of* ***controlling*** ***the negative effect of the COVID-19 virus*** *and promote* ***the patient's wellbeing***”.

## 4. The CONSULT system

**Figure 1:**
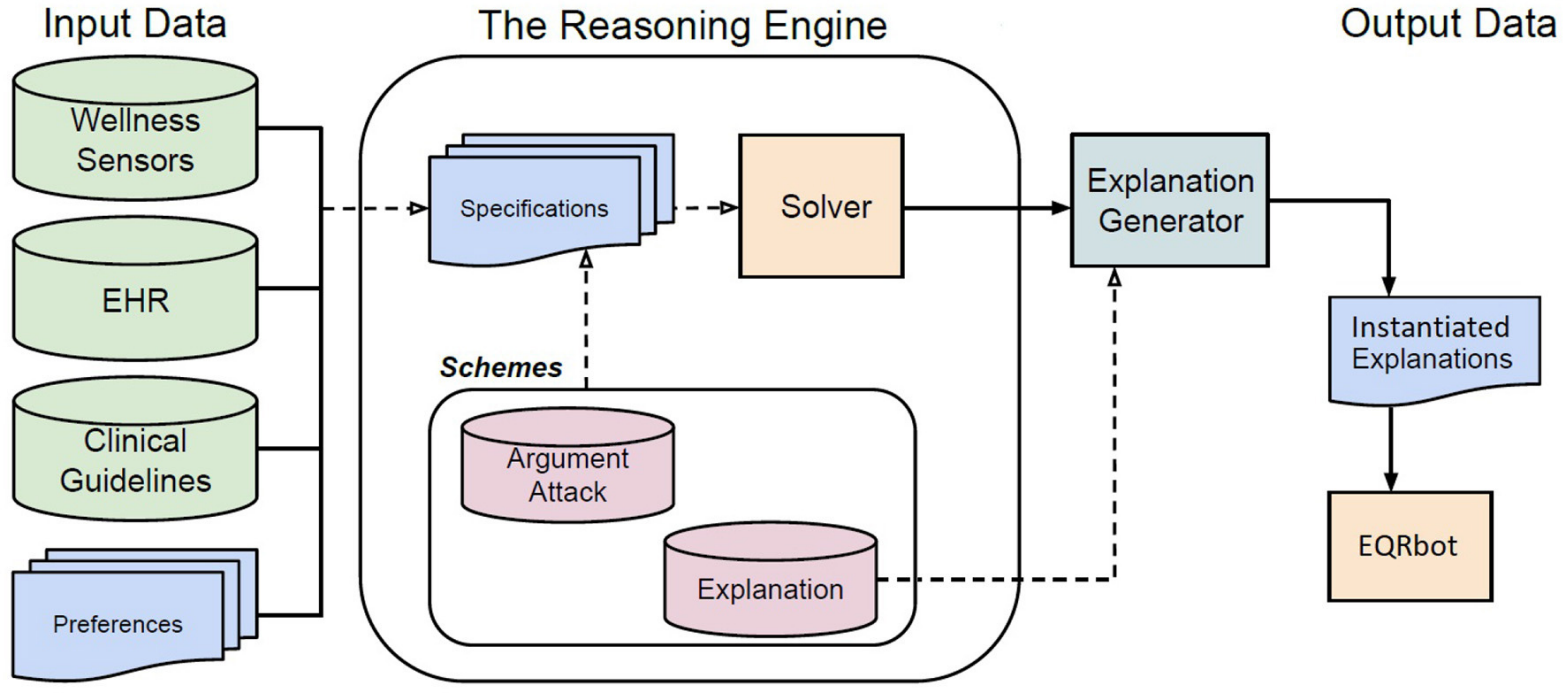
Flow chart describing the internal architecture of the CONSULT cDSS. The input data is provided by different sources. The *Schemes* are templates for structuring and representing arguments, attacks and explanations. A formal language (i.e., first-order logic) is used to encode the knowledge, retrieved from the input data, in terms of *Specifications* that will then instantiate the attack and argument schemes subsequently computed in the resulting AF by the ASPARTIX (Egly et al., 2008) *Solver*. The *Explanation Generator* [based on the sound and complete algorithm developed in Kökciyan et al. (2020)] constructs textual explanations for the recommendations according to explanation templates and the acceptable arguments produced by the *Solver*. The output will be stored in the *Instantiated Explanations* repository whose elements will feed the *EQRbot*, the chatbot responsible for interacting with the patient.

The CONSULT^7^ system is a novel data-driven mobile cDSS designed to help patients self-managing their condition and adhere to agreed-upon treatment plans in collaboration with healthcare professionals. Its main components are outlined in the following paragraphs and depicted in Figure 1. More details on the architecture of the system are available in Chapman et al. (2022).

### 4.1. Data inputs

There are three main types of data inputs into the CONSULT system: Wellness Sensors, Electronic Health Records and clinical guidelines. The *Wellness sensors* used included a Heart Rate monitor, a Blood Pressure Cuff and an ECG (Electrocardiogram) patch. The live parameters from these sensors are collected and displayed in one dashboard in the CONSULT system. This data is also used within the Argumentation Schemes instantiated in the reasoning engine. Information is additionally collected from the *Electronic Health Record* (*EHR*), for example the patients' known allergies and prescriptions along with their general medical history. Finally the *clinical guidelines*, i.e., official documents published by medical organizations (as the already mentioned NICE guidelines), are also represented within the system. The CONSULT system also considers the *preferences* of stakeholders allowing for personalized recommendations. Such preferences are rendered as hierarchies of information (e.g., values, treatment, and guidelines) elicited from multiple sources, e.g., patient and treating clinician (which also convey the interests of the healthcare organization and the ethical oath they have to observe). Ultimately, tailored algorithms are used to map these medical data and preferences into the formal language used by the reasoning engine. That is to say, stored in a knowledge base (i.e., the *Specifications*), data is represented in terms of facts and Answer Set Programming (ASP) rules using first-order logic.

### 4.2. Specifications

The EHR data provides information such as the *current_state* of a patient (including demographics and current medications), which need to be taken into account, along with the health parameters detected by the wellness sensors, when suggesting a treatment. Indeed, there may be age or other conditional restrictions related to the recommendation of, say, certain over-the-counter medications. For example, consider Frida, a pregnant patient currently suffering from fever and headache due to the COVID-19 virus. These facts will be formalized in first-order logic by the cDSS as *current_state*(fever, headache, COVID19) and *condition*(pregnancy). A treatment may then be recommended (as shown in Example 1) following the clinical guidelines of NICE-NG191^8^ and NHS^9^ (after their encoding into ASP-rules) that specifically handle those circumstances.

### 4.3. Schemes

Argument, attack and explanation schemes are templates representing common patterns of reasoning and relate a set of premises to a conclusion, all of which are sentences that can be represented in first-order logic and include variables that can be instantiated by data stored in a knowledge base. These schemes are kept in the *Schemes* repository and are rendered as ASP rules composed of a *rule body*, namely a conjunction of predicates (premises of the scheme), and a *rule head*, namely the scheme conclusion. The information stored in the *Specifications* data will then instantiate the elements of *Schemes* (i.e., attack and argument schemes) and thereupon will be fed to the *Solver*.

### 4.4. Solver and explanation generator

The argumentation-based reasoning engine runs on ASPARTIX (Egly et al., 2008), an ASP-*Solver* capable of computing arguments extensions under the required semantics (Dung, 1995). The reasoning engine leverages a formal representation of arguments through their respective argument schemes, critical questions and attacks to account for the conflicts between arguments in a given domain. The engine relies on the EvalAF algorithm to construct an argumentation framework for decision support and the ExpAF algorithm to provide explanations for acceptable arguments and attacks through the use of explanation templates^10^. The EvalAF algorithm generates an argumentation framework from a knowledge base and computes extensions under given semantics. The ExpAF algorithm maps acceptable arguments and attacks into explanations in natural language, using the sets of acceptable arguments and attacks, and corresponding explanation templates (Definition 3). In charge of the generation of such explanations is the sound and complete algorithm developed and implemented in Kökciyan et al. (2020).

### 4.5. Instantiated explanations

The *Instantiated Explanations* repository contains the rationales that justify the EQR explanation(s) (also member(s) of the repository) that serves as the pivotal element upon which all the other information is connected. Any answer to the questions moved by users of the CONSULT cDSS will be drawn from the data stored in such an archive. Notice that each explanation is tailored to the specific interacting patient's requirements, preferences and medical records. That is because the system manages only known information about the user and their conditions, thus providing suited routine recommendations conveniently retrieved by the applicable clinical guidelines (according to the predetermined cDSS resources and the patient's preferences). The user is made aware that CONSULT is not conceived to solve conflicts or handle unfamiliar data that would require professional medical expertise. Given this constraint, we can understand how the explanations stored within the *Instantiated Explanations* repository have to be finite.

## 5. EQRbot

**Figure 2:**
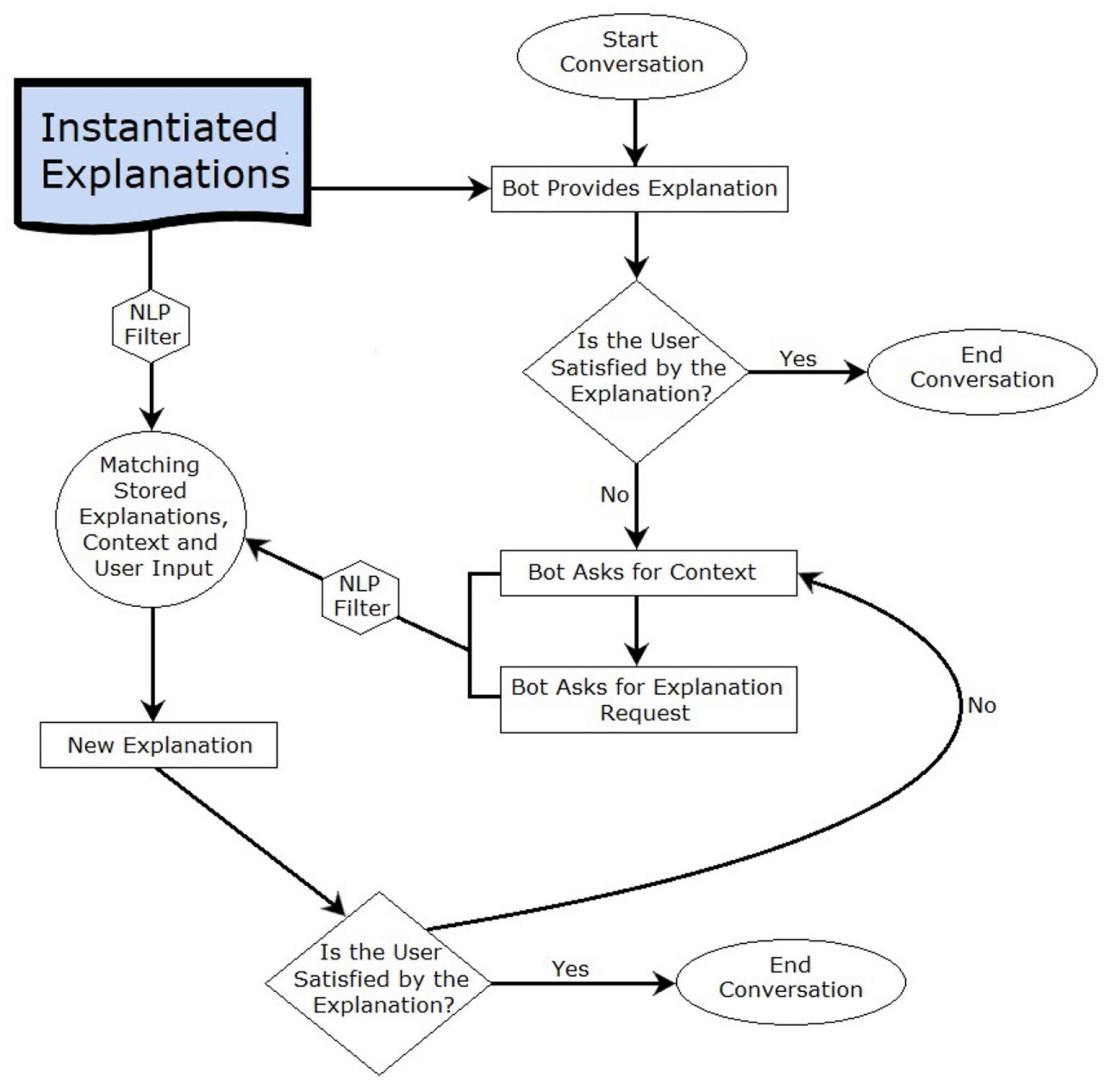
Flow chart describing the high-level operations performed by the chatbot (EQRbot).

The agent that will handle the interaction with the patient is a retrieval-type chatbot, i.e., a kind of bot that focuses on retrieving contexts and keywords from the user's prompts in order to select the best response to give.^11^ The explanation process will occur as delineated in Figure 2. After having provided the initial explanation (i.e., the EQR explanation informed by an acceptable instantiation of the EQR scheme), the patient will be asked to express their opinion. If the user is satisfied with the explanation, then the conversation will immediately end. Alternatively, the chatbot will demand: a brief context (e.g., “*Would you please specify the context of your explanation request?”*) along with the actual request from the patient. Consider that the interaction is not limited by a specific set of options to which the explainee needs to comply: the choice of words to use for formulating the inquiries is completely unrestricted. By matching stored explanations (all of which account for the stakeholders' preferences), context and user input, the bot will output the additional solicited information. Observe that the double query prompted by the conversational agent ensures a significant reduction of misunderstandings when providing answers to the patient. That is because the matching occurs *via* a double-layer word similarity counter function based on a BoW (Bag of Words) model. The explainer (chatbot) can be considered successful in its clarification attempt if the proposed explanation is deemed satisfactory by the user. Recall that the patient is aware of the EQRbot's inability to address questions regarding information not stored within the CONSULT system. As such, a satisfactory explanation may also be depicted as the realization that the user has to contact an healthcare professional should they have further queries. This will stop the loop of answers/questions and will end the conversation. It will continue otherwise.

It should be noted that the presence of multiple initial acceptable EQR explanations will not affect the chatbot operations. Since all of the explanations are acceptable, there is no need to further invoke the reasoning engine. The explanations are all considered equally good, seeing that our criteria for presenting an explanation is its acceptability (in turn influenced by the stakeholders' preferences), and so the EQRbot will randomly choose one of the available options and will then begin its interaction with the user. To this end, observe also that the bot is designed to avoid any unnecessary prolongation of the interaction to focus only on the required explanations. For this reason, the EQRbot will not start a conversation (nor even send a message) without the user prompt, but will react to each received text.

### 5.1. NLP filter

The chatbot employs a Natural Language Processing (NLP) filter in order to refine the input it receives from the patient and the stored instantiated explanations (Figure 2). The filtering process comprises: (a) the separation of the considered data into lists of single words (tokenization); (b) the elimination of the most common English words, including conjunctions and prepositions (stop-words removal); (c) the transformation of each word into its lemmatic form (lemmatization). The purpose of this refinement procedure is to ease the word matching between a patient's request and the system stored information. Notice that NLP does not influence the reasoning engine nor its outcome (i.e., the resulting arguments and their status), it only facilitates the matching operation.

### 5.2. The algorithm

The EQRbot's inner operations can be described by an algorithm, Algorithm 1, that takes as input the *Instantiated Explanations* repository (EXP), along with the set of all the possible user queries (Q) related to the data conveyed by the initially provided EQR_explanation (which is also an element of EXP). The procedure continues until the depletion of all the possible queries of Q, that is to say until the user is satisfied with the received information.

**Algorithm 1:** Matching Queries/Explanations.

**Input:** EXP, an EQR_explanation, and the (finite)
set of the possible user's queries Q
**Output:** all the requested explanations
1: print(EQR_explanation)
2: **for each** q ∈Q:
3: q == (c, r) *##* *q* *is a pair composed by a context (**c**) and specific request (**r**)* ##
4: *find_specific_explanation*(q)
5: **end for each**
6: ·
7: ·
8: **Function** find_specific_explanation(*q*)
9: *NLP_filter*(c)
10: *NLP_filter*(r)
11: specific_explanation = “ ”
12: similarity_counter = 0
13: provisional_explanation = “ ”
14: **for each** EX ∈ EXP\{EQR_explanation}
15: *NLP_filter*(EX)
16: **if** *double_layer_matcher*(c, r, EX) > similarity_counter **then**
17: similarity_counter = *double_layer_matcher*(c, r, EX)
18: provisional_explanation = EX
19: **endif**
20: **end for each**
21: specific_explanation = provisional_explanation
22: print(specific_explanation)
23: **end Function**

Intuitively, NLP_filter corresponds to the function that performs a series of Natural Language Process operations as outlined in 5.1. double_layer_matcher, instead, represents the BoW similarity procedure in charge of identifying the appropriate response to be delivered. double_layer_matcher takes advantage of the context designation, the frequency of key terms occurrence and multiple cross-counts of the input words and the system stored data. Each resulting explanation will then be printed and displayed in the chatbot graphical user interface (GUI).

Proposition 1. *Given the interacting user collaboration (i.e., no out-of-context, non-sense or out-of-the-system-capability input)*, *Algorithm 1* *is both sound and complete*.

Indeed, the procedure can provide the requested information that is correct according to the user's input (soundness), and all such answers can be conveyed by the algorithm (completeness). Obviously, this is limited by the data held by the system at the time of the explanation delivery. That is to say, the procedure can only generate explanations determined by the information saved in the system's knowledge base.

*Proof*.

- [Soundness] The chatbot retrieves the patient's prompt (q) as a pair of context (c) and request (r). Then, the function find_specific_explanation (lines 8–23) matches the input with one of the explanations stored in the system (EX) according to a BoW similarity procedure denoted double_layer_matcher (lines 16–18). The result of this operation will then consist of the information requested by the user. In case of a mismatch, the process can be repeated until the user's satisfaction (lines 2–5).
- [Completeness] All the requested information can be conveyed by the algorithm. Indeed, each additional explanation the patient might require (associated with the initial EQR explanation) is already saved in the system. They can all be retrieved with the corresponding query (lines 2–5).

Since no machine learning operation is involved, hence no time is consumed in training a model, the algorithm will take polynomial time to run. That is because the function find_specific_explanation will be called a maximum of |Q| times, i.e., up to the number of elements of Q.

### 5.3. Implementation

**Figure 3:**
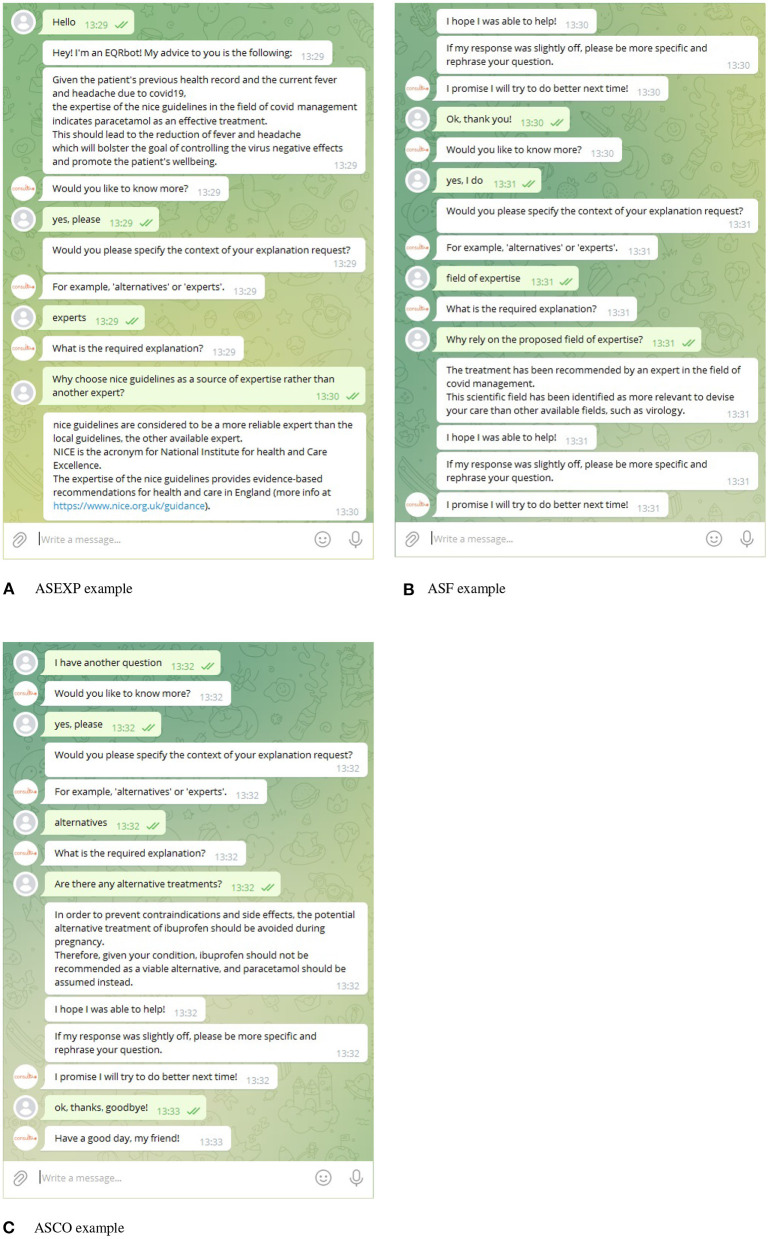
Instance of a conversation with the EQRbot starting from the explanation of Example 1. The displayed interaction captures the patient inquiries regarding the involved expert **(A)**, the field of expertise **(B)** and possible alternatives to the proposed treatment **(C)**. Matching the user's input, context and the information stored in the system, the EQRbot provides the additional requested explanation *via* the acceptable instantiations of the, respectively, ASEXP, ASF, and ASCO schemes.

Let us consider the EQR explanation of Example 1. We implemented it *via* a Telegram GUI. We chose to deploy the EQRbot *via* Telegram due to (i) its reputation as one of the most well-known and utilized instant messenger applications, and (ii) its programmer-friendly BOT API. To clarify the interaction depicted in Figure 3, let us suppose that the user monitored by the CONSULT system is, once again, Frida. The electronic health record supplies the cDSS with two pieces of information: the patient is pregnant, and she is currently suffering from fever and headache caused by the COVID-19 virus. To ease Frida from the pain, when prompted, the CONSULT reasoning engine computes an acceptable (as per Definition 1) piece of advice in the form of an EQR explanation. The EQRbot will display such a recommendation while encouraging also to ask for more details. Supplying the context and the specific request, the patient will demand the rationale behind the choice of the expert that provides the received clinical advice. The chatbot reply involves a natural language explanation based on the acceptable instantiation of the *AS for Expert Reliability* (Figure 3A). In the example, the system considers NICE guidelines as the most reliable source and provides an explanation accordingly. Notice, however, that CONSULT is engineered as a cDSS that supplies recommendations attained from general health guidelines (e.g., NICE). As explicitly stated before its usage, since the system is not supposed to handle conflicts that require professional medical knowledge to be solved, the users should seek advice from their general practitioners would such a circumstance occur. Indeed, this may cause significant harm to the patient if not handled correctly, as emphasized in Snaith et al. (2021). For the same reason, the cDSS (hence the EQRbot) is also updated by the patient's latest wellness sensor readings, the data in their EHR (so, for example, it will not recommend a therapy that has caused negative side effects in the past) and their preferences regarding treatments. The conversation continues in Figure 3B, where Frida interrogates the chatbot for additional information regarding the relevance of the selected field of expertise to the proposed recommendation. Similarly to its previous reply, the bot will formulate an explanation based upon the acceptable instantiation of the *AS for Relevant Field of Expertise*. To completely satisfy the patient's need for clarification, the chatbot will have to output one last explanation, this time about the acceptable instantiation of the *AS for alternative Clinical Options*. Indeed, the patient desires to know if alternative treatments are available (because, for instance, the drug indicated by CONSULT is not currently accessible to her). However, the cDSS confirms its previous recommendation informing Frida that, due to her pregnancy, paracetamol is the most appropriate remedy to assume (Figure 3C).

### 5.4. Evaluating the EQRbot against the CONSULT baseline

A seven day within-subjects mixed-methods run in-the-wild (Waterson et al., 2002) study has been conducted to assess the usability and acceptability of the CONSULT system with two different versions: with and without a chatbot. Such a pilot study demonstrated that real users could employ the application over an extended period (Balatsoukas et al., 2020). Connie, the conversational agent previously equipped with the cDSS at the time of the experiment, accommodates the patients willing to seek immediate evidence-based advice about a specific health problem. Informed by the user's vital data, preferences, EHR and clinical guidelines retrieved by the CONSULT system, the chatbot provides any additional explanation regarding the proposed recommendation. The main aspects that characterize Connie can be outlined as:

- *User's Input*. No free interaction occurs since the user's prompt is restricted to hard-coded multiple options.
- *Interface*. The chat, and related conversation log, are graphically displayed *via* Mattermost^12^.
- *Chatbot Type*. Connie is a rule-based chatbot^13^, i.e., an agent capable of responding only by following predetermined (scripted) replies according to the user's input.
- *Reasoning Engine*. The bot leverages the results of the operations performed by the CONSULT system by means of the computational argumentation solver ASPARTIX.
- *Explanation Delivery*. No particular strategy is deployed. The explanations are triggered *via* the options selected by the user.

**Figure 4:**
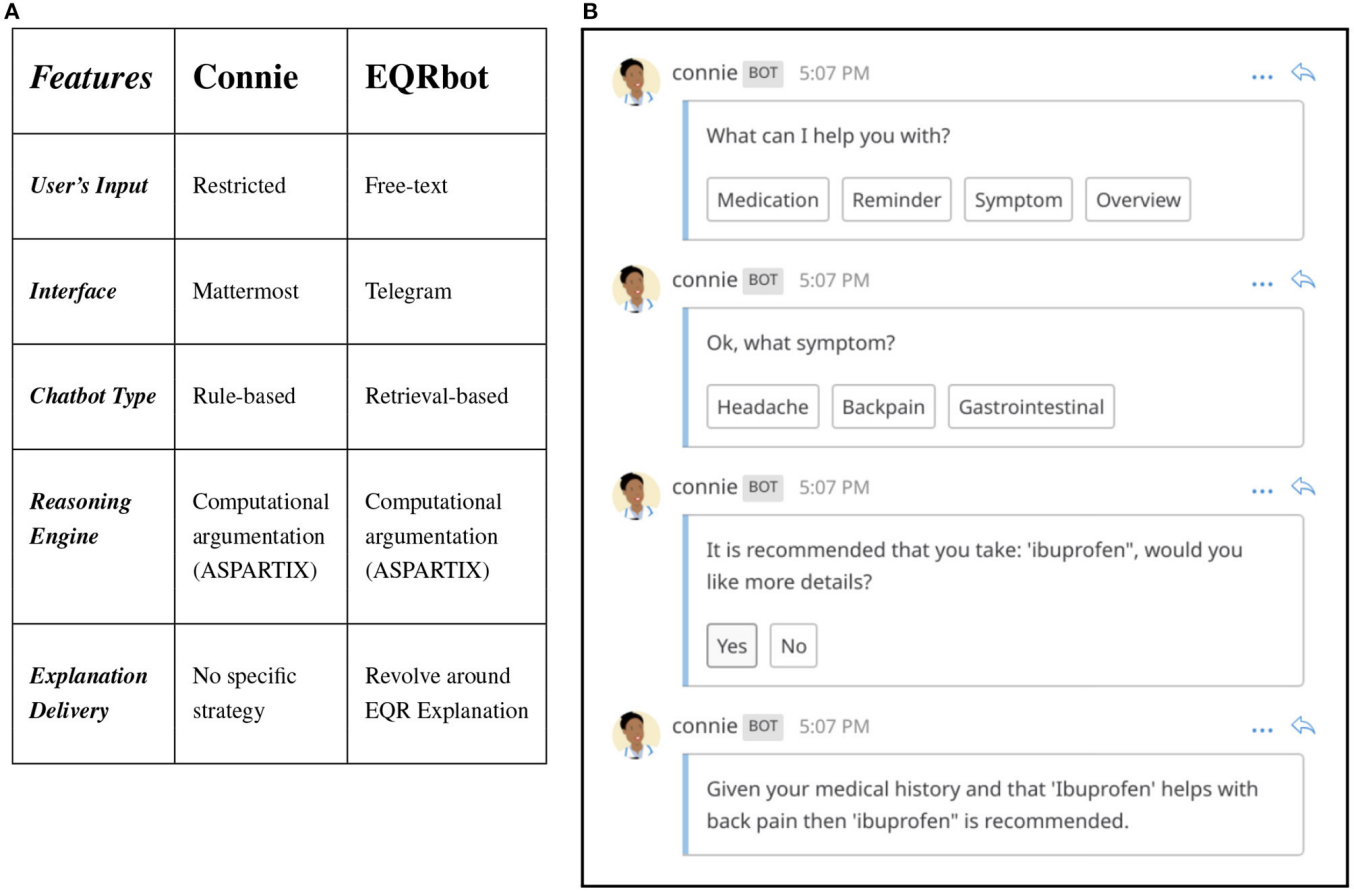
**(A)** Comparison of EQRbot and Connie's main features. **(B)** Example of interaction between a patient and Connie.

An example of a conversation with Connie is illustrated in Figure 4B. Here the interacting patient is given the choice of selecting among four different options in response to the question “*What can I help you with?”*. The user then decides to report a symptom concerning backpain, asking also for more details once a reply is given. This option triggers one last response from the chatbot, thus providing the explanation behind the rationale of the proposed recommendation. Nonetheless, Connie presents some limitations, as summarized by the result of the pilot study: “[…] *the lack of a more natural conversation flow when interacting with the chatbot (e.g., close to the one that they [the patients] would have with their GP)”* (Balatsoukas et al., 2020).

Against Connie, considered as the previous baseline, the EQRbot yields several advantages, as highlighted by the comparative table of Figure 4A:

- *User's Input*. Free textual interaction. Each user's prompt will be parsed by the chatbot NLP filter and matched with the most appropriate reply. Any non-sense or out-of-context input will be addressed by a random response from the bot.
- *Interface*. The chat, and related conversation log, are graphically displayed *via* Telegram.^14^
- *Chatbot Type*. EQRbot is a retrieval-based chatbot, i.e., an agent that mostly retrieves its replies from a database of potential responses according to the most relevant match with the user's input.
- *Reasoning Engine*. The bot leverages the results of the operations performed by the CONSULT system by means of the computational argumentation solver ASPARTIX.
- *Explanation Delivery*. The aim is to reduce the number of potential user queries (including possible follow-on questions) and concerns by concentrating the most relevant information about a specific recommendation within a single explanation, i.e., the one elicited by an acceptable instantiation of the EQR scheme.

The EQRbot represent an improvement over Connie since it addresses (in four out of the five listed main features) the shortcomings ensuing from the pilot study outcome. Indeed, it allows for *(i)* better approximations of natural conversations without textual restriction, by employing *(ii)* Telegram GUI, i.e., a more user-friendly, and popular messaging application than Mattermost. In general, *(iii)* retrieval-based chatbots are more versatile and flexible than rule-based ones, hence more suited for real-world exchange of arguments. Finally, despite its simplicity, *(iv)* having an explanation strategy bring the EQRbot closer to an authentic question-answer dialog.

## 6. Discussion

Although argument schemes-based clinical explanations have already been employed in studies such as Atkinson et al. (2006), Kökciyan et al. (2020), Shaheen et al. (2021), and Sassoon et al. (2021), the EQR scheme proposed herein emerges as a model designed to efficiently deliver a significant amount of information (both practical and theoretical) at once. Indeed, EQR explanations constitute the core notions around which all the data, possibly required by subsequent follow-on queries, are clustered into user-friendly natural language snippets of texts. Nevertheless, the envisaged implementation (of which Figure 3 represents a very restricted example) of this new argument scheme *via* the EQRbot presents some limitations, the most prominent of which concerns the delivery of the explanations. The conversation that occurs with the patient, albeit simple and clear, lacks a fully-fledged formal protocol with a complete set of available locutions, tracked utterances commitment store, precise semantics and pragmatics (McBurney and Parsons, 2009). A protocol for an explanation dialog has been given in Bex and Walton (2016) with a complete list of locutions. However, to evaluate the provided explanation, the explainee needs to resort to a different dialog protocol (denoted examination). Similarly, Madumal et al. (2019) devise a study for modeling explanation dialogs by following a data-driven approach. The resulting formalization embeds (possibly several) argumentation dialogs nested in the outer layer of the explanation protocol. Finally, also the dialog structure proposed (for a previous version of the CONSULT chatbot) by Sassoon et al. (2019) in the context of explanations for wellness consultation exploits multiple dialog types (e.g., *persuasion, deliberation* and *information seeking*) and their respective protocols whilst mostly focusing on the course of action to undertake. This is different from the anticipated EQR dialog (sketched in McBurney and Parsons, 2021 as Explanation-Question-Response), whose protocol is halfway between *persuasion, information-giving/seeking and query* and more comprehensively incorporates locutions for handling each of these tasks without the need for adopting a *control layer* (Cogan et al., 2006) or switching between protocols. This allows for a simpler formalization and more genuine dialogs. For all of these reasons, future implementations of EQRbot will provide for the addition of a formal protocol and an adjustment to the chatbot's memory. That is to say, the bot's capability for recalling the arguments previously moved in the conversation and recorded in the commitment store. Indeed, considering that the EQR explanations have been informed by several CQs that should comprehend all the possible challenges moved to them, no problem will arise if the user's inquiries regard these explanations or their specifics. However, if the inquiries concern a reference to an argument that occurred in an earlier stage of the dialog, the chatbot may not be able to properly address the request.

The landscape of argumentation-based chatbots has seen an increase in interest in recent years. For example, ArguBot (Bistarelli et al., 2021), developed using Google DialogFlow, employs ASPARTIX to compute arguments from an underlying Bipolar AF, or BAF, (Cayrol and Lagasquie-Schiex, 2005) to support or challenge the user's opinion about a dialog topic. The conversational capabilities of ArguBot are, however, restricted by the arguments stored in the BAF as its knowledge base, limiting its dialectical potential only to specific fully-developed interactions. One of the main problems concerning argumentation-based chatbots is indeed the creation of a proper knowledge base from which the bot's arguments can be retrieved and employed to interact with the user. The research of Chalaguine et al. (2018) and Chalaguine and Hunter (2018, 2019) outline *harvesting* and *crowd-sourcing* methodologies capable of collecting arguments and counter-arguments on a specific topic, thus generating suitable and persuasive knowledge bases for chatbots [e.g., Chalaguine and Hunter (2020), and, harnessing also hand-crafted counterarguments due to the topic sensitivity, Chalaguine and Hunter (2021)]. Unlike the studies presented thus far, the knowledge base of the EQRbot is personalized on the patient's preferences and health data. That information is constantly updated, making it possible to generate a potentially indefinite number of diverse explanations (although the user will need to restart the conversation to allow for the acquisition of the modified knowledge base, since the EQRbot cannot alter its stored responses during an interaction). Finally, although still resorting to similarity algorithms to retrieve appropriate arguments from a fixed knowledge base, Fazzinga et al. (2021) designed a bot that performs a reasoning step with multiple elements of user information before outputting each reply. Notice, however, that our EQRbot already performs such a step before selecting the final answer. Indeed, the list of responses fed to the chatbot is the result of a computation of the framework's acceptable arguments generated from the data and templates presented in the CONSULT system. Restarting the conversation with the EQRbot before each new explanation request will ensure that a new reasoning process (that involves the overall AF) will take place.

Lastly, further improvements could also arise by combining the recent developments in the field of *Argument Mining* (Cabrio and Villata, 2018) with additional chatbot code-based instructions. The swift generation of AFs comprising domain-specific arguments can indeed assist the bot in performing engaging dialogs such that the user's claims might be constructively challenged by more persuasive and precise explanations. The mining should occur from a specialized dataset composed of annotated clinical abstracts as in Mayer et al. (2020) or Stylianou and Vlahavas (2021), where the authors provide a complete argument mining pipeline capable of classifying argument components as *evidence/claim* and argument relations as *attack/support*. In addition, the research presented in Mayer et al. (2021) extends the pipeline by detecting also the effects on the outcome associated with the identified argumentative components.

### 6.1. Planned user study

To fully evaluate the EQRbot performances, we are currently planning a user study. The goal of the study is to analyze the interactions between the patients and the chatbot, such as how often a conversation is initiated, how long the question/answer session is on average and which are the most common queries prompted by the user. In particular, we are interested in a qualitative assessment of the provided explanations and the general level of users' satisfaction toward them. As discussed before, CONSULT handles data from patients' Electronic Health Records and suggests treatments (following clinical guidelines and stakeholders' preferences) that have already been tested on the interacting subjects, thus preventing any contraindications or side effects. Therefore the recommendations and potential explanations delivered by the EQRbot will not risk harming the user, and will instead indicate to contact medical professionals when required. However, if such a message occurs frequently, this may have the negative consequence of raising distrust from the patient against the system which may then overlook such a recommendation hence precluding (possibly essential) communications with the main caregivers. For this reason, the participants of the study will be preemptively informed of the cDSS limitations and its main functions. In addition, they will also receive a user manual to be examined whenever needed. The study is expected to last for two weeks, during which the patients are free to explore the system functionalities and interact with the chatbot. Before the beginning of the experiment, the participants will be interviewed in order to understand what they seek and prospect from the interactions with the cDSS and the EQRbot. A similar interview will also be conducted at the end of the study, where it will be possible to compare the user experience with their initial expectations and where feedback for further improvements will be collected.

## 7. Conclusion

Designed as a model capable of efficiently delivering both practical and theoretical information during inter-agent (human or AI) explanations, the EQR argument scheme proposed herein formalizes the consequences yielded (and the presumptive reasoning leading to them) by acting upon an expert opinion. In this paper, we outlined an approach that integrates the EQR scheme in the current research landscape involving decision support systems and argument-based explanations. In particular, we have focussed on studies regarding medical applications of such reasoning patterns, and we have presented a possible way of enhancing the related explanation templates. Indeed, one of the main advantages offered by the provided contributions is the incorporation of clinically specialized AS (e.g., ASPT) into the newly detailed EQR scheme structure. This will give more opportunities for inquiry to an agent seeking clarification since there are more aspects that can be interrogated and that can help in finding a satisfactory and more complete explanation. For example, which expert is informing the suggested treatment is a piece of information that might increase the patients' trust in the medical recommendation system. Furthermore, we have presented an implementation of the proposed contributions by equipping the CONSULT cDSS with a chatbot that employs acceptable EQR scheme instantiations as the core element to convey explanations. This is a substantial contribution to the research field of argumentation-based human-agent interactions. Indeed, our bot is guided exclusively by an argumentation reasoning engine in its decision-making process while it converses with the user: no machine learning algorithm is involved in the procedure. In addition, NLP is utilized only as a means for enhancing the word matching between the user input (which is completely free and not limited to multiple choice options) and the system stored explanations. Unlike other chatbots in the literature, the EQRbot depends upon a dynamic knowledge base that is constantly updated by the patient's data received from the health sensors and their EHR. This entails more personalized and, possibly, disparate interactions, as long as the user restarts the conversation (which will allow the reasoning engine to generate new explanations upon the updated knowledge base). Finally, we deploy our bot *via* Telegram. Such a choice ensures a convenient programmer API along with a well-known and user-friendly GUI.

## Data availability statement

The provided link: https://github.com/FCast07/EQRbot refers to the GitHub repository that stores the chatbot programming code.

## Author contributions

FC: main idea, first draft, and chatbot implementation. AG: telegram GUI for the chatbot. PM, SP, and IS: conceptualization, edit, and review. ES: edit and review. All authors contributed to the article and approved the submitted version.

## Footnotes

^1^In Dung (1995), the author employs Reiter's Default logic (Reiter, 1980) and Pollock's Inductive Defeasible logic (Pollock, 1987) as an example of non-monotonic reasoning rendered *via* abstract argumentation.

^2^ https://www.who.int/publications/who-guidelines

^3^ https://www.nhs.uk/medicines/amoxicillin/

^4^ https://www.nhs.uk/medicines/cefalexin/

^5^ https://www.nhs.uk/medicines/azithromycin/

^6^ https://www.nice.org.uk/guidance

^7^ https://consultproject.co.uk

^8^NICEcovid-managementguide section that specifically covers fever-management.

^9^NHSwebpage section that specifically deals with ibuprofen assumption risks during pregnancy (redirected from NICE webpage).

^10^ https://git.ecdf.ed.ac.uk/nkokciya/explainable-argumentation

^11^ https://github.com/FCast07/EQRbot

^12^ https://mattermost.com/

^13^ https://www.codecademy.com/article/what-are-chatbots

^14^ https://telegram.org/
